# Efficacy of subcutaneous Levothyroxine in a case of refractory hypothyroidism: A case report

**DOI:** 10.1097/MD.0000000000029690

**Published:** 2022-06-30

**Authors:** Annabelle Naman, Brigitte Delemer, Didier Marot, Elise Michelet, Bénédicte Decoudier, Sara Barraud

**Affiliations:** a Centre Hospitalier Universitaire de Reims, Service d’Endocrinologie – Diabète – Nutrition, Reims, France; b Université de Reims Champagne-Ardenne UFR de Médecine, Reims, France; c CRESTIC EA 3804, Université de Reims Champagne Ardenne, UFR Sciences Exactes et Naturelles, Reims, France; d Centre Hospitalier Universitaire de Reims, Pôle de Biologie Territoriale, Service de Biochimie-Pharmacologie-Toxicologie, Reims, France; e Centre Hospitalier Universitaire de Reims, Service Pharmacie, Reims, France.

**Keywords:** hypothyroidism, levothyroxine, refractory hypothyroidism, subcutaneous

## Abstract

Daily oral synthetic levothyroxine (LT4) is the main treatment for hypothyroidism, which, in most cases, allows the regression of symptoms and the normalization of the thyroid function. However, rarely, despite a high dose of oral LT4, hypothyroidism persists and is called refractory hypothyroidism. Intravenous or intramuscular treatment is then often necessary. We report the case of a patient with refractory hypothyroidism successfully treated with subcutaneous LT4.

**Interventions and outcomes:**

After 4 weeks of weekly intravenous injections of 200 µg LT4 in complement to the oral treatment, thyroid balance was improved (TSH: 21.8 mIU/L). We tested the replacement of intravenous with subcutaneous injections of LT4 and gradually increased injection frequency from 1 to 3 injections per week (600 µg/week). Simultaneously, oral treatment was gradually tapered off, and within a few months, thyroid function tests were normalized. Two years later, hormone levels remained normal without symptoms of hypothyroidism. The only side effect was a local reaction in the first few weeks of injections, which spontaneously resolved.

**Lessons:**

In this case of unexplained oral LT4 malabsorption, subcutaneous injection allowed a self-administrated physiological dose of LT4 3 times weekly. Considering the efficacy of subcutaneous injection of LT4, this treatment could be a safe and easy alternative for patients with malabsorption.

## 1. Introduction

Hypothyroidism is a common condition, mainly due to autoimmune thyroiditis and thyroidectomy.^[1]^ The first-line treatment is daily oral synthetic levothyroxine (LT4). In the majority of cases, this treatment allows the regression of symptoms and the normalization of the thyroid function. But in rare cases, despite high dose of LT4 (usually ≥ 1.9 µg/kg/day), hypothyroidism persists; it is named refractory hypothyroidism and can be related to malabsorption, drug interference, or poor compliance (also called pseudomalabsorption).^[2]^ We report the case of a patient with refractory hypothyroidism successfully treated with subcutaneous LT4.

## 2. Case report

### 2.1. Patient concerns and diagnosis

A 53-year-old woman was referred to our center for persistent severe autoimmune hypothyroidism for 8 years. She presented periorbital myxedema, hoarse voice, pale skin, hair loss, asthenia, weight gain (+ 30 kg), and slow thinking. Despite 250 µg LT4, thyroid function tests showed: TSH 79 mIU/L (0.27–4.2), free thyroxine (FT4) 4.4 pmol/L (11.5–22.7), free triiodothyronine (FT3) 2.01 pmol/L (3.08–6.47).

Over 2 years, we progressively increased oral LT4 up to 400 µg (4.4 µg/kg/day) and associated it with 75 µg/day of Liothyronine (Cynomel®, Sanofi), without reaching euthyroidism (TSH: 40 mIU/L, FT4: 9.3 pmol/L). Switching to another oral LT4 formulation or bedtime intake was not effective.

Due to suspected drug interaction, Carbamazepine (severe epilepsy) was discontinued without TSH level improvement. The usual causes of malabsorption, gastric atrophy, coeliac disease, and Helicobacter Pylori infection could be ruled out. Therefore, gastroscopy with gastric and duodenal biopsy was performed. No antiparietal cell or anti-transglutaminase antibodies were found.

Following the absorption test (administration of 1000 µg LT4 and a 6-hour follow-up), there was an insufficient increase in FT4 level: 6.82 to 9.01 pmol/L (0.53–0.7 ng/dL).

### 2.2. Interventions and outcomes

Given the inefficacy of oral treatment, weekly intravenous injections of 200 µg LT4 (L-thyroxine Serb®) were added to the oral treatment, requiring weekly hospitalization. After 4 weeks, thyroid balance was improved (TSH: 21.8 mIU/L).

To improve quality of life and avoid hospitalization, we evaluated the feasibility of replacing intravenous with subcutaneous injections. In France, the only LT4 parenteral formulation available is the above-mentioned labeled for intravenous or intramuscular injection and is provided as a 1 ml ampoule containing 200 µg of LT4. The subcutaneous treatment was initially carried out under medical supervision and combined with oral LT4. The injection was performed without dilution of the product, with a 25 Gauge needle of 16 mm length, using the “pinch-up” technique and a 45° angle. The injection frequency of LT4 was increased gradually from 200 µg (1 injection) to 600 µg (3 injections) per week, corresponding to a dose of 0.92 µg/kg/day. Simultaneously, oral treatment was gradually tapered off over 3 months, and the patient was educated to perform injections at home.

**Figure 1.**
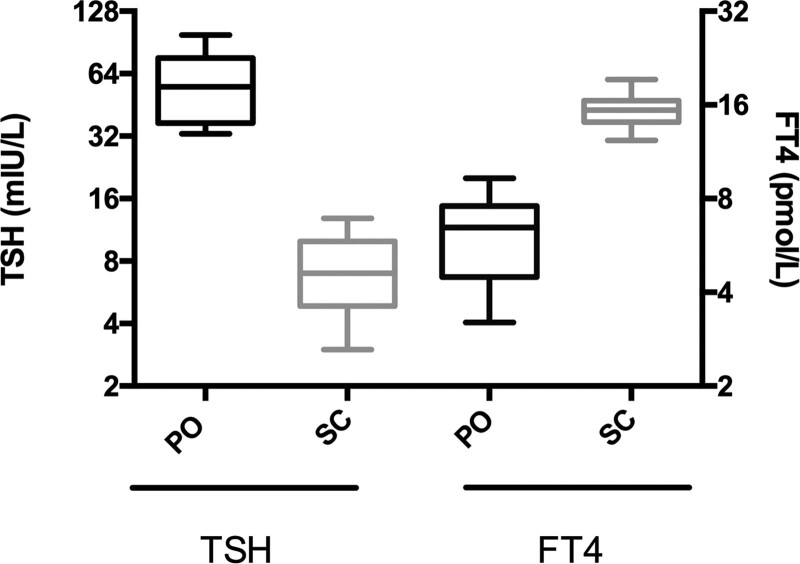
Comparison of thyroid function with oral or subcutaneous Levothyroxine (with oral Levothyroxine, median TSH was 55.4 mIU/L (n = 6) and median FT4 was 6.5 pmol/L; with subcutaneous Levothyroxine, median TSH was 7 mIU/L (n = 6) and median FT4 was 14.4 pmol/L. Reference ranges: TSH: 0.27–4.2 pmol/L, FT4: 11.5–22.7 pmol/L). FT4 = free thyroxine), PO = *per os*, SC = subcutaneous.

In 2 ½ months, thyroid function tests were normalized. Two years later, hormone levels remained normal, and symptoms of hypothyroidism have completely disappeared (Fig. 1). The only side effects were injection site pain and localized mild erythema in the first few weeks of injections, which spontaneously resolved.

## 3. Discussion

In this case of unexplained oral LT4 malabsorption, subcutaneous injection allowed a self-administrated physiological dose of LT4 3 times weekly, with good tolerance.

The patient presented refractory hypothyroidism, but the investigations failed to find a formal cause. She was treated with Carbamazepine, but the TSH level remained high after switching to another antiepileptic drug. The regular intake of soybeans could have contributed to the difficulties of absorption of LT4. A test of absorption was carried out with FT4 determination to detect pseudomalabsorption. The interpretation of LT4 absorption tests remains controversial. Some calculate LT4 uptake from total thyroxine (TT4).^[3]^ Other teams focus on FT4, whose change is well correlated with TT4, and different interpretations have been proposed..^[4–7]^ Whatever the thresholds used, the increase in FT4 of our patient remained insufficient, in favor of true malabsorption.

To simplify at-home treatment, off label subcutaneous administration route was carried out with parenteral LT4. The main risk was a local side effect, but this seemed limited, given the small volume injected and the injection site rotation.^[8]^ Furthermore, this administration route avoids the risk of overdose, inherent to weekly intravenous injection, and the unexpected variations in digestive absorption of a high oral dose.

To our knowledge, 3 other cases of LT4 subcutaneous administration have been reported. First, Jauk et al showed a good efficacy of this treatment with a normalization of the thyroid function.^[9]^ Unfortunately, after 6 months, a local reaction occurred, leading to the discontinuation of the treatment and the return to a more restrictive intravenous treatment. The formulation used was not specified; so we cannot assess the role of the excipients and the injected volume in the occurrence of this side effect. Subcutaneous treatment was also used for a second patient, but finally, the intramuscular route was preferred due to local reaction.^[10]^ The formulation of LT4 is not reported in this article either. The last case describes the weekly administration of L-Thyroxin Henning inject® 500 µg in a patient with malabsorption.^[11]^ The sample has a volume of 10 ml, which was divided into 2 to favor a better tolerance. This treatment allowed normalization of the thyroid function, and after 7 months, it was still well tolerated. Subcutaneous administration, therefore, seems to be effective, but the question of local tolerance remains, which may depend on the formulation used.

## 4. Conclusions

In this case of unexplained oral LT4 malabsorption, subcutaneous injection allowed a self-administrated physiological dose of LT4 3 times weekly. Considering the efficacy of subcutaneous injection of LT4, this treatment could be a safe and easy alternative for patients with malabsorption, on the condition of a good local tolerance.

## Acknowledgment

Thank you to SERB for graciously providing the treatment to the patient.

## Author contributions

Data curation: Sara Barraud.

Writing – original draft: Annabelle Naman, Brigitte Delemer, Sara Barraud.

Writing – review & editing: Brigitte Delemer, Sara Barraud, Bénédicte Decoudier, Didier Marot, Elise Michelet.

Literature search: Annabelle Naman, Sara Barraud.
